# Global Dynamics of Porcine Enteric Coronavirus PEDV Epidemiology, Evolution, and Transmission

**DOI:** 10.1093/molbev/msad052

**Published:** 2023-03-03

**Authors:** Hao Zhang, Chuangchao Zou, Ouyang Peng, Usama Ashraf, Qiuping Xu, Lang Gong, Baochao Fan, Yun Zhang, Zhichao Xu, Chunyi Xue, Xiaona Wei, Qingfeng Zhou, Xiaoyan Tian, Hanqin Shen, Bin Li, Xiangbin Zhang, Yongchang Cao

**Affiliations:** State Key Laboratory of Biocontrol, Life Sciences School, Sun Yat-sen University, Guangzhou, China; State Key Laboratory of Biocontrol, Life Sciences School, Sun Yat-sen University, Guangzhou, China; State Key Laboratory of Biocontrol, Life Sciences School, Sun Yat-sen University, Guangzhou, China; State Key Laboratory of Agricultural Microbiology, Huazhong Agricultural University, Wuhan, China; Guangdong Provincial Key Laboratory of Malignant Tumor Epigenetics and Gene Regulation, Sun Yat-sen Memorial Hospital, Sun Yat-sen University, Guangzhou, China; State Key Laboratory of Biocontrol, Life Sciences School, Sun Yat-sen University, Guangzhou, China; Key Laboratory of Zoonosis Prevention and Control of Guangdong Province, College of Veterinary Medicine, South China Agricultural University, Guangzhou, China; Institute of Veterinary Medicine, Jiangsu Academy of Agricultural Sciences, Key Laboratory of Veterinary Biological Engineering and Technology, Ministry of Agriculture, Nanjing, China; State Key Laboratory of Biocontrol, Life Sciences School, Sun Yat-sen University, Guangzhou, China; State Key Laboratory of Biocontrol, Life Sciences School, Sun Yat-sen University, Guangzhou, China; State Key Laboratory of Biocontrol, Life Sciences School, Sun Yat-sen University, Guangzhou, China; State Key Laboratory of Biocontrol, Life Sciences School, Sun Yat-sen University, Guangzhou, China; Guangdong Enterprise Key Laboratory for Animal Health and Environmental Control, Wen's Group Academy, Wen's Foodstuff Group Co. Ltd, Yunfu, China; Guangdong Enterprise Key Laboratory for Animal Health and Environmental Control, Wen's Group Academy, Wen's Foodstuff Group Co. Ltd, Yunfu, China; Guangdong Enterprise Key Laboratory for Animal Health and Environmental Control, Wen's Group Academy, Wen's Foodstuff Group Co. Ltd, Yunfu, China; Guangdong Enterprise Key Laboratory for Animal Health and Environmental Control, Wen's Group Academy, Wen's Foodstuff Group Co. Ltd, Yunfu, China; Institute of Veterinary Medicine, Jiangsu Academy of Agricultural Sciences, Key Laboratory of Veterinary Biological Engineering and Technology, Ministry of Agriculture, Nanjing, China; Jiangsu Co-innovation Center for Prevention and Control of Important Animal Infectious Diseases and Zoonoses, Yangzhou University, Yangzhou, China; Guangdong Enterprise Key Laboratory for Animal Health and Environmental Control, Wen's Group Academy, Wen's Foodstuff Group Co. Ltd, Yunfu, China; College of Animal Science, South China Agricultural University, Guangzhou, China; State Key Laboratory of Biocontrol, Life Sciences School, Sun Yat-sen University, Guangzhou, China

**Keywords:** coronavirus, porcine epidemic diarrhea virus, epidemiology, evolution, transmission

## Abstract

With a possible origin from bats, the alphacoronavirus Porcine epidemic diarrhea virus (PEDV) causes significant hazards and widespread epidemics in the swine population. However, the ecology, evolution, and spread of PEDV are still unclear. Here, from 149,869 fecal and intestinal tissue samples of pigs collected in an 11-year survey, we identified PEDV as the most dominant virus in diarrheal animals. Global whole genomic and evolutionary analyses of 672 PEDV strains revealed the fast-evolving PEDV genotype 2 (G2) strains as the main epidemic viruses worldwide, which seems to correlate with the use of G2-targeting vaccines. The evolving pattern of the G2 viruses presents geographic bias as they evolve tachytely in South Korea but undergo the highest recombination in China. Therefore, we clustered six PEDV haplotypes in China, whereas South Korea held five haplotypes, including a unique haplotype G. In addition, an assessment of the spatiotemporal spread route of PEDV indicates Germany and Japan as the primary hubs for PEDV dissemination in Europe and Asia, respectively. Overall, our findings provide novel insights into the epidemiology, evolution, and transmission of PEDV, and thus may lay a foundation for the prevention and control of PEDV and other coronaviruses.

## Introduction

Porcine epidemic diarrhea (PED) is a highly contagious, acute intestinal disease caused by the Porcine epidemic diarrhea virus (PEDV). The onset of the disease is acute and rapid, and associated with clinical symptoms such as severe diarrhea, vomiting, and dehydration. PEDV can infect pigs of all ages; however, infected suckling piglets may exhibit mortality up to 100% (Jung et al. 2015). PEDV is an enveloped, single-stranded, positive-strand RNA virus that belongs to the genus *Alphacoronavirus* of the family Coronaviridae (Kocherhans et al. 2001). The PEDV genome is approximately 28 kb in length and comprised of a 5′ cap, a 3′ poly-A tail, 5′ and 3′ non-coding regions, and at least 7 open reading frames encoding for four structural and 17 non-structural proteins (Kocherhans et al. 2001; Lee 2015). The PEDV spike (S) protein is a surface immunogenic protein that mediates virus entry into the host cells and elicits the induction of antibody responses (Lee 2015).

PEDV was first detected in the United Kingdom in 1971 (Wood 1977), and then, rapidly spread to other European countries (Debouck and Pensaert 1980). Since 1980, PEDV has become prevalent in Asian countries, and has been considered as a threat to the pig industry (Takahashi et al. 1983; Chen et al. 2008; Puranaveja et al. 2009). Due to low disease incidence and mortality, PEDV has not gained considerable attention in the past; however, the emergence of variant PEDV strains of high virulence in China in 2010 has led to an increase in mortality rate of up to 100% (Chen et al. 2011). Later, in April 2013, the outbreak of PED in the United States and neighboring countries (Canada and Mexico) caused the death of >8 million piglets in the US alone (Vlasova et al. 2014; Ojkic et al. 2015).

In China, PEDV was first isolated in 1984 (Xuan et al. 1984). Since then, several epidemiological studies have reported the circulation of PEDV in China. The PEDV incidence was 80% in sows, 90% in fattening pigs, and 100% in suckling pigs in 1987 (Li and Zhong 1987). Afterward, the overall incidence saw a slight decrease. According to Du et al. (2004), an incidence of 42% with 5.69% mortality was summarized in 2004 on the farms of Guangxi province. Zhang et al. (2019) reported a positivity of 49.58% in samples collected during 2011–2014 from 29 provinces. These studies suggest that PEDV is extensively circulating in the pig population of China.

Before 2010, the PEDV burden in China has been sporadic due to the availability of inactivated or attenuated PEDV vaccines. However, such vaccines remained ineffective against variant PEDV strains which caused devastating losses to the Chinese pig industry in 2010 (Sun et al. 2012). Studies also suggest that Chinese PED cases displayed infections by strains from other countries, which has significantly facilitated the emergence of variant PEDV strains and their potential for pandemic outbreaks (Sun et al. 2015). A recent study by He et al. (2022) focused on the reconstruction of the geographical dispersal and the identification of factors influencing the PEDV spread in China. However, all analyses shown in the study were based on pig-trade data and the Sanger sequencing of PEDV *S1* gene, hence providing limited information. Therefore, large-scale and detailed studies of the molecular epidemiology and evolution of PEDV are urgently required.

Herein, we identified PEDV as a major virus in diarrheal pig samples collected from 2011 to 2021. Using genomic, evolutionary, and phylogeographic approaches, we unraveled important aspects of PEDV evolution and epidemiology such as source population, genetic recombination, time of origin, evolutionary rate, and dispersal history, which could be applicable for the control and prevention of PEDV and other coronaviruses.

## Results

### PEDV Epidemic, Sequencing, and Virus Isolation in China

**Fig. 1.**
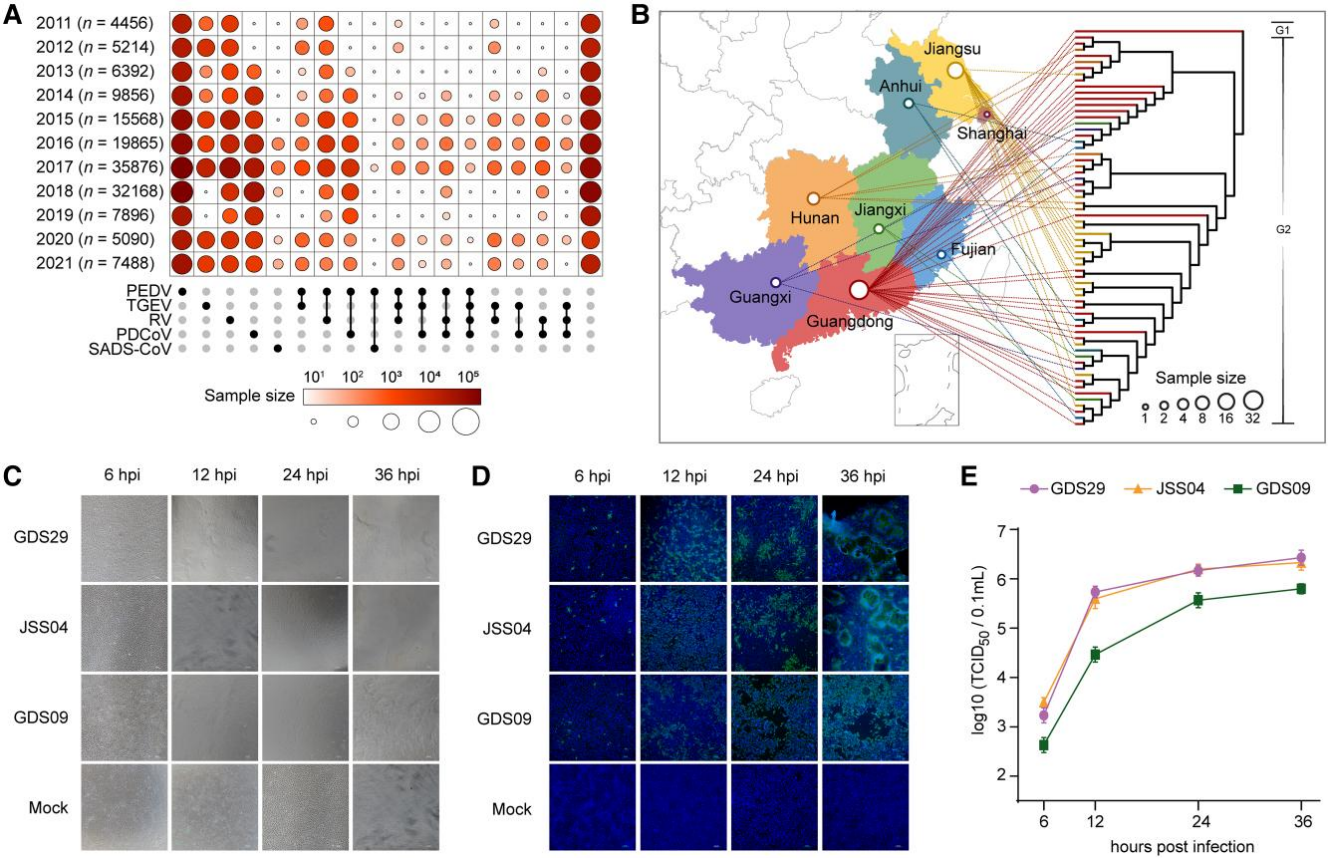
Characteristics of epidemics of PEDV from 2011 to 2021 in China. (*A*) A schematic representation of 149,869 fecal and intestinal tissue samples of pigs collected from the year 2011 to 2021. The upset plot arranged five common swine diarrhea-causing viruses: PEDV, TPEV, RV, PDCoV, SADS-CoV. The color shades and the size of circles represent the yearly sample size. (*B*) Phylogeographic distributions of 65 whole genomes sequenced PEDV strains from the year 2011 to 2021 in the study. The topological tree was built with the maximum likelihood method and 1,000 bootstrap replicates. The branch of the strain GDS09, belonging to the G1 genotype, is depicted in black in the phylogenetic tree. The size of circles in the provinces represents the sample size of sequenced PEDV full genomes. (*C*) Cytopathic effects of Vero-E6 cells infected with three representative PEDV strains (GDS29, JSS04, and GDS09) at 6, 12, 24, or 36 h postinfection (hpi), scale bar indicates 100 μm. (*D*) Immunofluorescence staining of the PEDV S protein in infected Vero-E6 cells. Cells were fixed at 6, 12, 24, or 36 hpi, and S protein was detected by indirect IFA. Nuclei were shown by 40,6-diamidino-2-phenylindole staining. The images of cells were acquired by a fluorescence microscope (Nikon Eclipse 80i), scale bar indicates 100 μm. (*E*) Viral titers in the culture supernatants were determined by TCID_50_ assay. Data are shown as mean ± SEM and are representative of three independent experiments.

We collected 149,869 clinical samples of feces and intestinal tissues of pigs that presented PEDV illness-like clinical symptoms from seven provinces and Shanghai City in China for 11 years (2011–2021) (fig. 1*A* and supplementary table S1, Supplementary Material online). These pigs showed obvious diarrhea, vomiting, weight loss, dehydration, dead piglets, and intestinal epithelial villi damage (supplementary fig. S1*A* and *B*, Supplementary Material online). The symptoms were associated with infection mainly caused by PEDV, transmissible gastroenteritis virus (TGEV), rotavirus (RV), porcine deltacoronavirus (PDCoV), or swine acute diarrhea syndrome coronavirus (SADS-CoV), as assessed by virus isolation in these samples (supplementary fig. S1*C*, Supplementary Material online). Among them, PEDV was found as a major causative agent with a constant infection-positive rate of >40% across the period of 11 years (supplementary fig. S1*C*, Supplementary Material online). RV and PDCoV showed a lower (1–20% and 0–14% respectively) but stable infection rate across the timeline (supplementary fig. S1*C*, Supplementary Material online). Of the total samples, a proportion of 3.21% exhibited co-infections of PEDV, TGEV, RV, PDCoV, or SADS-CoV, while 31.28% of samples remained undiagnosed (fig. 1*A* and supplementary table S2, Supplementary Material online). We further sequenced 74,568 confirmed PEDV-positive samples and obtained 65 whole PEDV genome sequences (supplementary fig. S1*D* and *E*, Supplementary Material online). The sequenced genomes were named based on the geographical region of sample collection and were preliminarily genotyped according to the evolutionary tree (fig. 1*B* and supplementary table S3, Supplementary Material online). Among them, only one PEDV strain (GDS09) was genotyped as G1 and the rest 64 strains were G2 genotype. We next isolated and cultured three PEDV strains, namely GSD09 (G1 subtype), GDS29 (G2 subtype), and JSS04 (G2 subtype), that showed replication kinetics in cultured Vero-E6 cells at 6, 12, 24, and 36 h infection time-points (fig. 1*C*–*E*). The proliferative capacity of GSD09 was noticed lower than that of GDS29 and JSS04, as determined by cytopathic effects, staining of the PEDV S protein-positive cells, and viral titers in the supernatants (fig. 1*C*–*E*).

### Homology, Evolution, Inter- and Intra-Regional Recombination Analysis of PEDV Strains

**Fig. 2.**
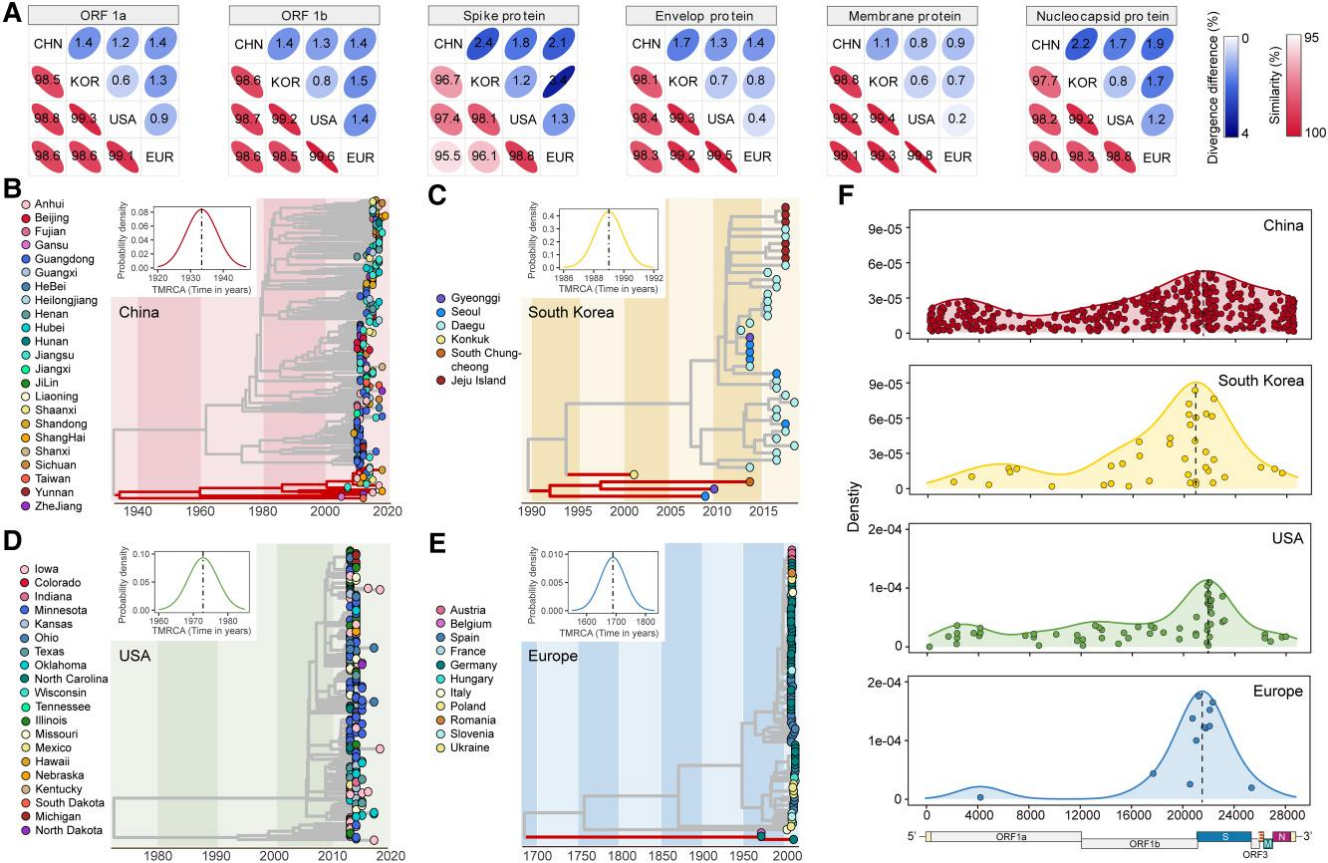
Evolution and recombination of PEDV strains of four geographical regions (China, South Korea, the US, and Europe). (*A*) The similarity and divergence of nucleotide sequences of six PEDV genes (ORF1*a*, ORF1*b*, spike, envelop, membrane, and nucleocapsid) among geographical region pairs. (*B*–*E*) Maximum likelihood time-scaled phylogenetic trees of full PEDV genomes of four geographical regions: (*B*) China, (*C*) South Korea, (*D*) the US, and (*E*) Europe. The branches of G1 PEDV strains are highlighted. Different marks of the dots represent different administrative regions or countries. The *x*-axis represents the time in years. The inner plots showed the PP densities of the most recent common ancestor (TMRCA) of four geographical regions. (*F*) Breakpoint distributions of recombination events in PEDV strains of four geographical regions. PP, posterior probability.

Sequence analysis of 672 complete PEDV genomes, sequenced in this study (*n* = 65) or obtained from public resources (*n* = 607), indicated highest diversity in the S gene (supplementary table S4 and supplementary fig. S2*A*, Supplementary Material online). We next calculate the strain numbers and found that the US, China, Europe, and South Korea are the top four areas. To find whether there were differences between these areas, a comparative analysis of sequence homology between PEDV sequences isolated from four geographical regions (China, South Korea, the US, and Europe) was implemented, which revealed a markedly higher sequence divergence between Chinese isolates and the isolates of the other three regions (fig. 2*A*).

The root-to-tip distances and the most recent common ancestors of four geographical regions were also estimated using Treetime program (fig. 2*B*–*E* and supplementary fig. S2*B*–*E*, Supplementary material online). The common ancestor of PEDV strains in China could be dated before 1940, whereas it could be dated to the early 1700s in Europe. The estimated common ancestors of PEDV strains in South Korea and the US were relatively closer to the year 2000. Furthermore, we compared the rate of evolution in the PEDV genomes obtained from all four geographical regions (supplementary fig. S2*B*–*E*, Supplementary Material online). The results showed that the evolution rate of PEDV isolates in terms of substitution per site per year was highest in South Korea (7.75 × 10^−4^), moderate in China (2.36 × 10^−4^) and the US (1.65 × 10^−4^), and lowest in Europe (5.33 × 10^−5^). The same patterns of evolutionary rates were also shown using BEAST2 program (supplementary table S5, Supplementary Material online).

Since PEDV is a coronavirus, it is prone to genetic recombination (Kim et al. 2015). Using the SplitsTree program, no recombination event was found between PEDV and other coronaviruses, while many recombination events were observed among the PEDV strains (supplementary fig. S3*A*, Supplementary Material online). In addition, we investigated the recombination of 65 sequenced strains and other strains from three geographical regions (South Korea, the US, and Europe) (supplementary fig. S3*B*–*D*, Supplementary Material online). No recombination events were found between sequenced strains from our study and other regions (supplementary fig. S3*B*–*D*, Supplementary Material online). Furthermore, we analyzed the genetic recombination in the PEDV genomes taken from the four geographical regions using the RDP5 program. In all isolates, the recombination events were observed to be localized in the genomic region spanning from the nucleotide position 18,000–24,000. Precisely, the recombination breakpoints were mainly localized in the head and body of the S gene for American, Chinese, and European isolates. However, the South Korean isolates showed recombination at the junction of ORF1 and S genes (fig. 2*F* and supplementary table S6, Supplementary Material online). We also counted the number of recombination events in the genomes of wild-type and vaccine PEDV strains obtained from all four geographical regions. The genomic sequences of both wild-type and vaccine isolates from China showed the highest number of recombination events (supplementary fig. S4*A*–*E* and supplementary table S6, Supplementary Material online). For instance, the strain CN/Liaoning25/2018 is likely to have originated through the recombination of vaccine isolate SQ2014 of the G1 genotype and the wild-type strain GDS07 of the G2 genotype (supplementary fig. S4*B*, Supplementary Material online). Overall, these data demonstrate that the PEDV is evolving faster in South Korea but undergoing the highest recombination in China than in the other geographical regions.

### Haplotype Dynamics of PEDV

**Fig. 3.**
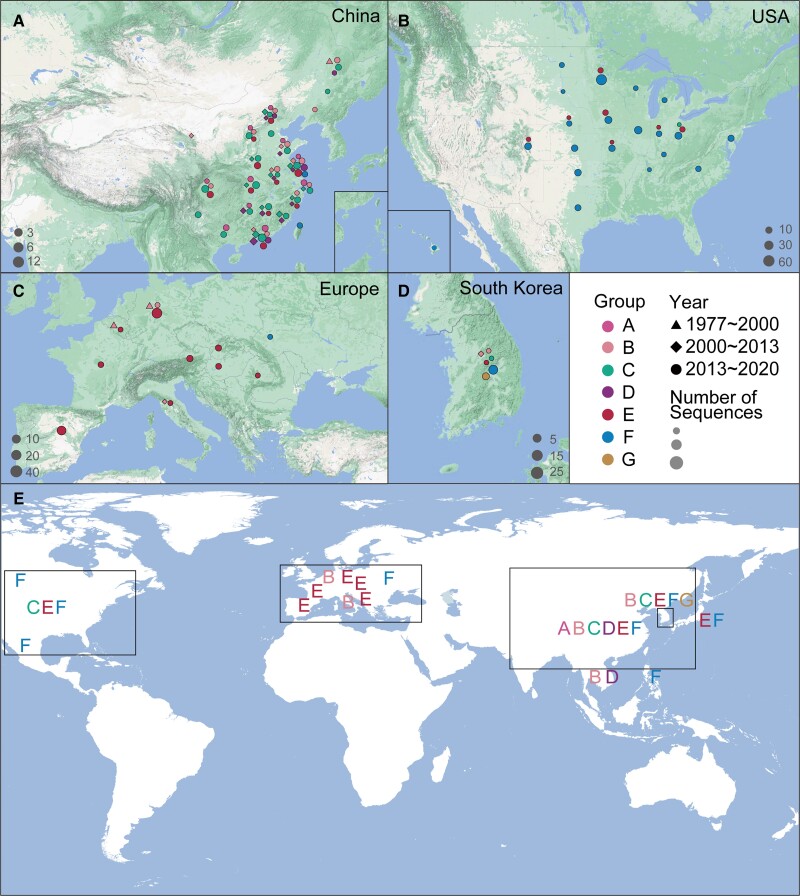
Haplotype dynamics of PEDV. Seven groups (*A*–*E*) of haplotypes were identified in supplementary figure S6, Supplementary Material online. (*A*–*D*) NSP8 haplotype distribution in (*A*) China, (*B*) the US, (*C*) Europe, and (*D*) South Korea. (*E*) Global distribution of various haplotype groups.

To analyze the time and genetic relationship of the genetic evolution of PEDV in the region, we used the nucleotide sequence of the non-structural protein NSP5, NSP8, and NSP12 of PEDV for haplotype analysis (supplementary figs. S5–S7, Supplementary Material online). NSP8 is a conserved gene in coronavirus which interferes with protein trafficking to the cell membrane upon infection (Zhang et al. 2018; Banerjee et al. 2020). As shown in figure 3 and supplementary figure S6, Supplementary Material online, a total of 156 haplotypes of PEDV strains could be divided into 7 large haplotype groups (Groups A–G). Viral strains in four geographical regions were distributed as follows: Chinese strains in groups A, B, C, D, E, and F, American strains in group F, European strains in group E, South Korean strains in groups B, C, E, F, and G. In terms of time (year), PEDV strains were introduced in 2013 in the US, 2000 in South Korea, 1997–2000 and 2013 in Europe, and 2000 in China. In addition, the haplotype analysis of two other PEDV conserved genes, NSP5 and NSP12 (Li, Ma, et al. 2020), was performed. A total of 212 haplotypes of NSP5 and 356 haplotypes of NSP12 were observed, among which, approximately 50% originated from China. Hence, these analyses suggest that the haplotype dynamics are higher in China than that in other geographical regions.

### Spatiotemporal Reconstruction of PEDV Dissemination Across the World

**Fig. 4.**
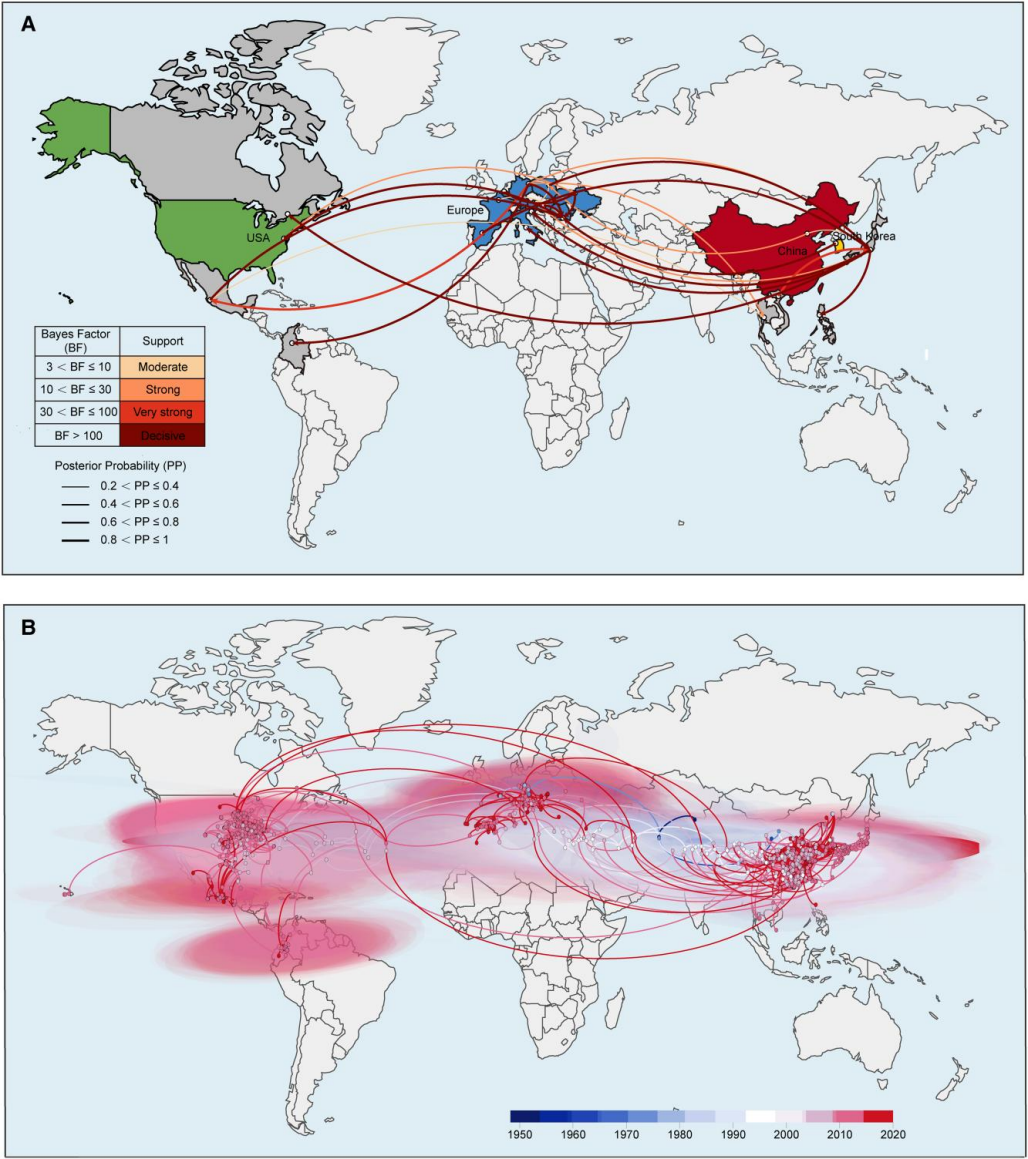
Spatiotemporal diffusion of PEDV strains around the world. (*A*) The discrete phylogeographic analysis was performed with the Bayesian stochastic search variable selection approach. We displayed the intensity of the estimated transition events associated with a BF support higher than three. The thickness of the dissemination links represented the PP. (*B*) Continuous phylogeographic analysis was performed with the lognormal RRW diffusion model. We mapped the MCC tree and 95% highest posterior density regions based on trees subsampled from the post-burn-in posterior distribution of trees. Nodes of the tree are scaled. A 95% highest posterior density regions were computed for successive time layers, superimposed using the same scale reflecting time cropped using worldwide international borders.

To explore the spatiotemporal spread route of PEDV across the world, the BEAST software was employed. The discrete phylogeographic model results showed that Europe, Asia, and the Americas are the major hubs of the virus dissemination across the world with a strong dissemination link found between Europe and Asia and between Europe and the Americas (fig. 4*A*). Among them, Germany is the export center of PEDV in Europe, and the export locations include Poland, Romania, Japan, Thailand, Mexico, and Colombia. Romania is the import center of PEDV in Europe, with sources including six countries like Germany. In Asia, Japan is found as the import and export center of PEDV. The import sources include China, Vietnam, Germany, Poland, and Spain, whereas the export destinations include the Philippines, Italy, Slovenia, Romania, Austria, and Canada (fig. 4*A*). The results of the continuous phylogeographic model showed that PEDV spread mainly in Europe in the early stage, then it was introduced to Asia and remained broadly epidemic in China, South Korea, and other Asian countries. After that, PEDV began to spread in the US and other American countries, and until now it is still widespread across the world (fig. 4*B*). The estimated effective population sizes over time showed that PEDV went through two rapid expansion waves in 2010 and 2013 around the world, then the population size fluctuated at a relatively high level (supplementary fig. S8*A*, Supplementary Material online).

The estimated effective population sizes of PEDV over time in China showed that there was a rapid expansion of the virus in 2010. Since then, the population size remained at a high level (supplementary fig. S8*B*, Supplementary Material online). Further analysis of a discrete phylogeographic model of Chinese regions suggested that the virus dissemination mainly occurred from North to South, where Jiangsu and its surrounding provinces, such as Guangdong, Guangxi, Hunan, Hebei, and Shandong showed strong dissemination links. Hubei demonstrated the properties of the PEDV import and export hub (supplementary fig. S9*A*, Supplementary Material online) in China, exhibiting strong dissemination links with 11 provinces. These results were similar to that as derived from the continuous phylogeographic model (supplementary fig. S9*B*, Supplementary Material online).

Collectively, these analyses provide the basis for PEDV dissemination around the globe and could be beneficial in regulating trading rules across borders.

### Genotyping Dynamics of PEDV

**Fig. 5.**
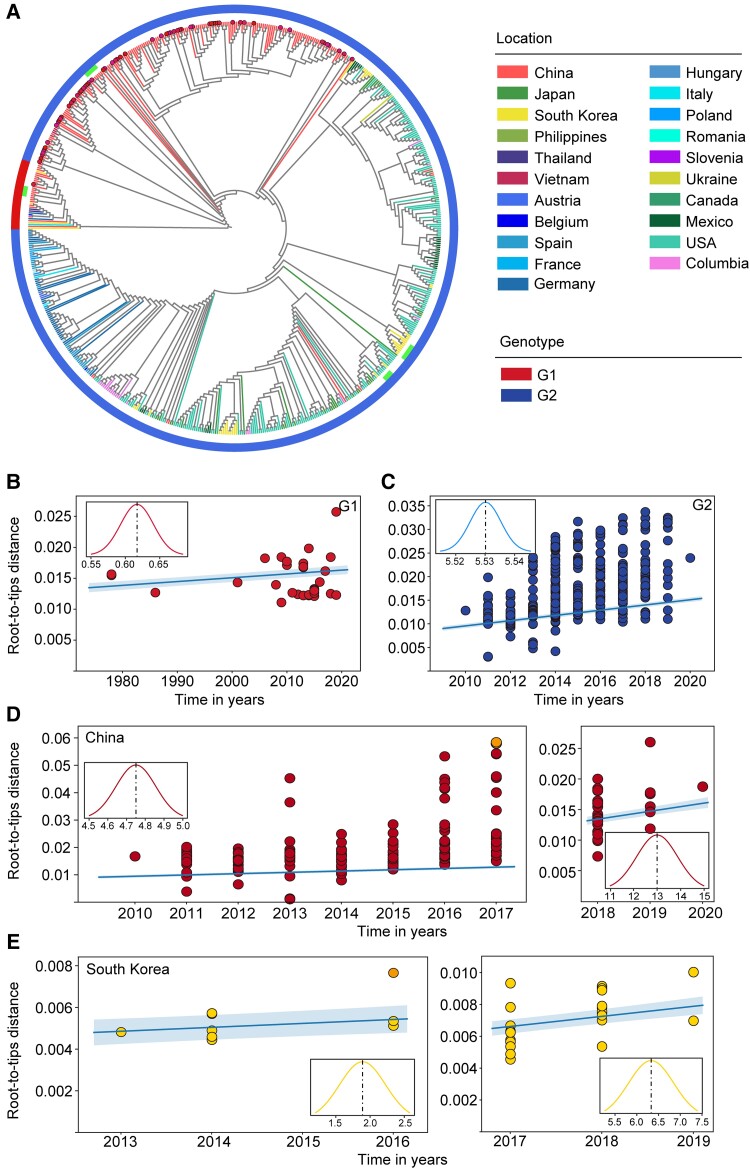
Phylogenetic analysis of complete PEDV genomes (*n* = 672) and estimated evolutionary rate of PEDV strains. (*A*) Phylogenetic tree constructed from 672 complete PEDV genomes using ML method. The outside layer denotes the genotypes. The highlighted dots indicate 65 PEDV strains sequenced in this study. The extra bars indicate the clusters of wild vaccine strains and vaccine-like strains. (*B*–*E*) Root-to-tip distance plots on sampling date and the estimated evolutionary rates of PEDV strains. (*B*) Strains (*n* = 33) of G1 genotype around the world, (*C*) strains (*n* = 639) of G2 genotype around the world, (*D*) Chinese strains of G2 genotype collected before the year 2018 (*n* = 173) and after the year 2018 (*n* = 34), and (*E*) Korean strains of G2 genotype collected before the year 2017 (*n* = 13) and after the year 2017 (*n* = 24). The highlighted strains in the outer plots were excluded in the linear fitting and evolutionary rate estimation according to TreeTime (Sagulenko et al. 2018). The inner plots show the posterior probability densities of the mean estimated evolutionary rate and the *x*-axis represents the evoltionary rate (×10^−4^ substitutions per site per year).

We next assessed the genotyping and spatiotemporal pattern of dissemination using phylogenetic analysis approaches. All 672 PEDV strains were classified into G1 and G2 genotypes, which exhibited a long evolutionary distance from each other (fig. 5*A*). Most strains were of G2 genotype with a proportion of 100%, 95.6%, 90.2%, and 89.9% in the US, Europe, South Korea, and China, respectively. The proportion of G1 strains was highest in China (10.1%) followed by South Korea (9.8%) and Europe (4.4%), whereas no such genotype strain was detected in the US, which may indicate that the burden of G1 strains is steadily increasing in China (supplementary fig. S10, Supplementary Material online). In addition, the phylogenetic analysis revealed that G2 strains distributed in China and Europe were evolutionarily divergent from each other. In contrast, the American and South Korean G2 strains clustered together in evolutionary tree (fig. 5*A*). To further compare evolutionary differences between G1 and G2 strains, we analyzed their evolutionary rates by considering all globally available sequences to date. It was observed that the evolutionary rate of G1 strains (6.17 × 10^−5^ substitutions per site per year) was lower than the G2 strains (5.53 × 10^−4^ substitutions per site per year) (fig. 5*B* and *C*). We then calculated the evolutionary rate of the G1 strains in China and G2 strains in the US, Europe, China, and South Korea (supplementary fig. S3*C*, supplementary fig. S11*B*–*D*, and supplementary table S5, Supplementary Material online), and the results were analogous to those as seen with global evolutionary rate analysis.

Given that vaccines are important means of virus prevention and control and that both China and South Korea have marketed commercial PEDV vaccines (Park et al. 2018; Li, Yang, et al. 2020), we next asked whether the use of such vaccines has impacted the PEDV evolution in both countries. Analysis of PEDV sequences available before and after the marketing of vaccines revealed that vaccines have greatly increased the rate of virus evolution both in China (before: 4.75 × 10^−4^ substitutions per site per year and after: 1.30× 10^−3^ substitutions per site per year) and South Korea (before: 1.89 × 10^−4^ substitution per site per year and after: 6.33 × 10^−4^ substitution per site per year) (fig. 5*D* and E), considering G2 strains only. We also used BEAST program to calculate all these evolutionary rates and found similar results (supplementary table S5, Supplementary Material online).

Overall, these data indicate that G2 strains are evolving faster than the G1 strains globally, and this phenomenon may associate with PEDV vaccine usage.

### Protein Structure Analysis of PEDV Genotypes G1 and G2 Strains

**Fig. 6.**
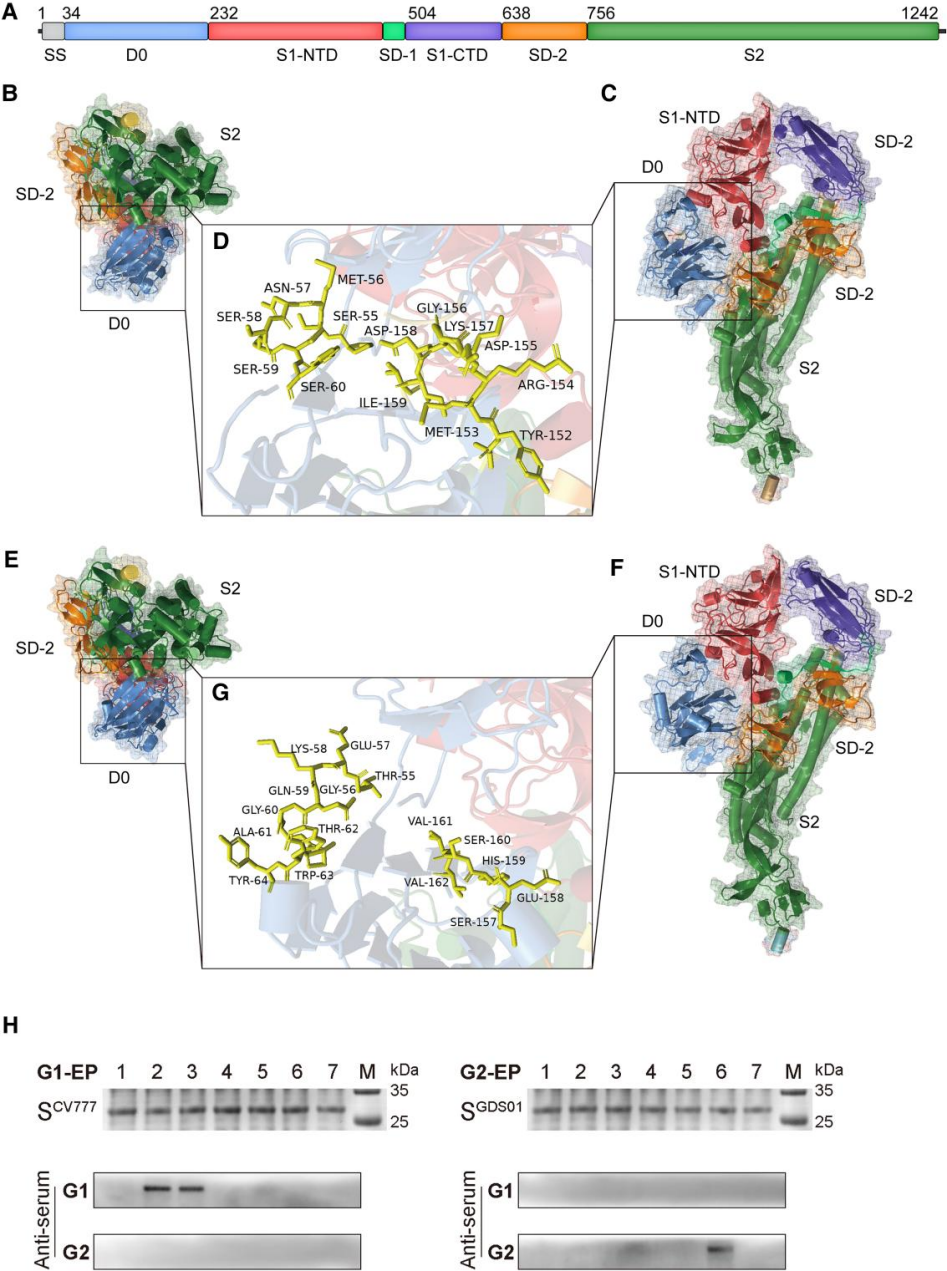
Comparison of the spike protein of genotype G1 and G2 viruses. Strains CV777 (Genbank accession No., AF353511) and JSS04 (Genbank accession No., MW143083) were used as representative strain of genotypes G1 and G2. (*A*) Schematic of PEDV spike protein. (*B*–*D*) Cartoon representation of amino acid sequences of the spike protein of CV777 with different perspectives. (*D*) Amino acid sequences at positions 55–60 and 152–159 of the spike protein of CV777. (*E*–*G*) Cartoon representation of amino acid sequences of the spike protein of JSS04 with different perspectives. (*G*) Amino acid sequences at positions 55–64 and 157–162 of the spike protein of JSS04. (*H*) Identification and validation of PEDV genotype-specific epitopes.

Considering that G2 strains are epidemic in China that the G1 subtypes are still spreading in China, we assessed the genotype distribution of all 672 PEDV strains. It was found that the proportion of G1 subtypes is highest in China (10.1%), followed by Korea (9.8%) and Europe (4.4%). However, no G1 subtypes were detected in the US (supplementary fig. S10, Supplementary Material online). As the head region of S gene showed the greatest diversity and recombination events (supplementary fig. S2, Supplementary Material online and fig. 2*F*), we then compared the amino acid sequence of S protein of a few representative G1 and G2 subtypes. The obvious differences were localized at the D0 domain ranging from the amino acid positions 55–64 and 157–164 (fig. 6*A* and supplementary fig. S12, Supplementary Material online). The strain of the G2 genotype (JSS04) exhibited amino acid insertions and substitutions at both sites when compared to the strain of the G1 genotype (CV777). We further simulated the protein structures of these variable sites using PyMOL, which revealed noticeable structural differences at both sites (fig. 6*B*–*G*). When compared to CV777, an increase of *α*-helix at position 55–64 and a replacement of *α*-helix with *β*-turn at position 157–164 were observed in JSS04.

Since the sera positive for the G1 or G2 genotype cannot be cross-recognized (Wang et al. 2016), we asked whether variable sites in S protein bear key residues for genotype-specific antibody recognition. To this end, we recombinantly expressed short peptides harboring these variable sites individually and incubated them with the sera positive for either the G1 or G2 genotype. It was observed that residues at position 55–64 were specific for the recognition of G1 strains, whereas residues at position 157–164 showed specificity to G2 strains (fig. 6*H*).

Altogether, these findings highlight the structural differences between PEDV genotypes, which might aid in understanding PEDV transmission and evolution.

## Discussion

Here, we performed a multicenter sampling of 149,869 fecal and intestinal tissue samples of diarrheal pigs, and identified PEDV as the most dominant virus in over the past 11 years in China with a prevalence rate of 61.8%, which is consistent with previous studies (Tian et al. 2021; Zhang et al. 2021; Li et al. 2022). We detected PEDV only in diseased pigs, indicating that PEDV is likely a pivotal etiologic factor of porcine diarrhea in China. Our 65 PEDV whole genomes could not only be utilized as a resource but also provide accessibility to the genomes of three newly discovered PEDV strains (GDS09, GDS29, and JSS04). A comparison of our sequenced genomes with published genomes highlighted that Asia had a more diverse and complex PEDV population compared to the US and Europe. Furthermore, the PEDV strains from South Korea and China exhibited highest recombination frequency and fastest evolutionary rate compared to the strains of other regions. It is known that PEDV was not reported in the US, Africa, and Australia until 2013 (Jung 2016). The highly pathogenic G2 strains emerged in China in 2010 and were transmitted back to Europe and America (Scott et al. 2016); however, it is still elusive when and how the G1 strains recombined with G2 strains. Considering these facts and our findings that all our 65 sequenced genomes showed recombination events, it would not be surprising to see the emergence of a novel genotype in Asian countries (fig. 3). He et al. (2022) revealed a possible association between swine trade and the spread of PEDV. According to their trade routes, the United States is the main source of PEDV introductions into other countries, while China could be an important hub of PEDV recombination because it imports most from across the world (He et al. 2022). Therefore, the large importation of piglets could be a possible reason for the most diverse PEDV population in China. These data are in agreement with our haplotype analysis, in which both the US and Europe possessed three haplotypes of the virus, whereas China harbored six haplotypes and the highest count. Compared with previous G1/G2 classification, our scheme provides a moderate system to define the linages that correlates with the PEDV spatiotemporal distribution. To our knowledge, this is the first haplotype study on PEDV, and our seven clades can be utilized as a quick map to guide the PEDV virus clustering worldwide.

Since PEDV is currently divided into genotypes G1 and G2 (Chen et al. 2013), our study calculated and compared the evolutionary rates of these two genotypes for the first time and found that the G2 strains are evolving faster than the G1 strains. This observation coincides with a study suggesting that the ongoing convergence of SARS-CoV-2 lineages includes multiple mutations that can enhance the persistence of diverse virus lineages during host immune recognition (Martin, Weaver, et al. 2021). Furthermore, the high evolutionary rate in G2 strains was associated with PEDV vaccine usage which is concordant with the enhanced fitness of Marek's disease virus strains under an imperfect vaccination regime (Read et al. 2015), and thus, could be a potential reason for the low efficacy of current PEDV vaccines (Park and Shin 2018). Therefore, future studies are needed to continuously monitor the PEDV evolution and to develop vaccines that could provide better protection.

We performed a Bayesian discrete phylogeographic inference to test the contribution of predictors to the spread of PEDV. In doing so, we confirmed the genetic distance between G1 and G2 strains. Indeed, the G1/G2 phylogenetic tree originated distinctly, which strengthened our model reliability. The results implied a relatively independent lineage within Europe and China, while the US and other areas showed interactive distribution. This correlated to the He et al. (2022) study which indicated that after the first PEDV dissemination from China, the United States is the main source of PEDV G2 introduction events in other countries. By employing the spatiotemporal reconstruction, Germany is the export center of PEDV in Europe, whereas Japan is the import and export center of PEDV in Asia. The simulation of these transmission routes is different from the results of He et al. (2022), possibly because they relied on PEDV *S1* gene sequence for simulation. In comparison, we employed PEDV whole genome sequences, which could better restore the real situation of the virus transmission and evolution, as known for the SARS-CoV2 epidemic evolution (Lemey et al. 2020; Viana et al. 2022). These analyses might be implied while trading the pig and pig-related products, and hence, could be useful to prevent the dissemination of PEDV across the borders.

Vaccine for classical G1 strains is protective; however, there is no new adaptive vaccine that could protect against the emerging G2 strain (Opriessnig et al. 2017; Yu et al. 2022). Our data provided a new insight into discovering the possible antigen sites that may lay a foundation for designing new vaccines. We indicated that the residues at position 55–64 were specific for the recognition of G1 strains, whereas the residues at position 157–164 showed specificity to G2 strains (fig. 5*C*). These data were confirmed by the reactivity of serotype-specific clinical serum samples. Therefore, it seems rational to design a gene vaccine that could target both G1 and G2 genotypes.

In summary, our study contributes to the understanding of PEDV biogeography. The results highlighted Asian countries as hubs for PEDV rapid evolution and recombination. We identified a novel virus haplotype G in South Korea, which could be an early warning for the creation of PEDV prevention and control policies. The US and Europe retained relatively stable virus strains; however, the United States is the main disseminator of the virus, which suggests that reasonable international epizootic practice still needs to be organized. Though, the fact that the limited access to WGS data of many other countries (such as Vietnam) limited our analysis, additional information from such countries could improve our understanding of the current phylogeographic map of PEDV. Hence, our study could be an important reference for the prevention and control of PEDV and other coronaviruses.

## Materials and Methods

### Sample Collection, Virus Isolation, and Sequencing

A total of 149,869 samples of feces and intestinal tissues of pigs, presenting PEDV illness-like clinical symptoms (supplementary table S1, Supplementary Material online), were collected from farms located in seven provinces (Jiangsu, Anhui, Jiangxi, Fujian, Hunan, Guangxi, and Guangdong) and Shanghai City of Southeast China from 2011 to 2021. PEDV was isolated using Vero-E6 cells as described previously (Hao et al. 2014). Viral pathogens responsible for pig diarrhea were detected by reverse transcription-polymerase chain reaction (RT-PCR) through virus-specific primers (supplementary table S7, Supplementary Material online). Vero-E6 cells were grown in Dulbecco's Modified Eagle Medium (DMEM; Gibco) supplemented with 10% (v/v) fetal bovine serum (FBS; Gibco) and 1% (v/v) penicillin-streptomycin. PEDV isolation was confirmed by an RT-PCR and an indirect immunofluorescence assay (IFA). RNAs from plaque-purified viruses were extracted using the QIAamp Viral RNA Kits (Qiagen). The cDNA was synthesized using random primers and the RevertAid First Strand cDNA Synthesis Kit (Thermo Scientific). Each sample was first screened by RT-PCR followed by viral isolation/purification and subsequently sequenced using the Illumina HiSeq 2500 platform with paired-end reads of 150 bp (PE150). The short reads of each sample are assembled by de novo following filtering out the host sequence. All 65 sequences have been deposited in the GenBank database (supplementary table S3, Supplementary Material online).

### Sequence Analysis

All (except attenuated strains) PEDV genome sequences, publicly available up to June 30, 2022, were retrieved from NCBI Genbank (https://www.ncbi.nlm.nih.gov/). Sequences of two PEDV vaccine strains (JP [Genbank accession No., MC425617] and KR [Genbank accession No., LY411674]) were excluded in the following analysis except in the recombination analysis (supplementary table S4, Supplementary Material online). Finally, the resultant 607 PEDV complete genome sequences and 65 newly sequenced PEDV genomes in this study (supplementary table S4, Supplementary Material online) were used for subsequent analyses. All 672 sequences were aligned using the MUSCLE v3.8.31 (Edgar 2004).

### Phylogenetic and Evolutionary Dynamic Analysis

The phylogeny of all 672 genomes was generated by adopting the maximum likelihood (ML) approach in the FastTree v2.1.7 (Price et al. 2010). The ML tree was constructed using the general time reversible (GTR) model of nucleotide substitution with a gamma (Γ) model of rate heterogeneity and 1,000 bootstrap replicates. The genotype of each virus strain was determined through the phylogenetic tree. The nucleotide sequence diversity at each given site was calculated as described previously (Zhao et al. 2020). Only the site containing ≤10% ambiguous bases (N) or gaps (−) was included to calculate nucleotide sequence diversity. Similar parameters were employed to calculate the similarity and divergence of the nucleotide sequences of polyproteins (ORF1a and ORF1b) and structural proteins (spike, envelope, membrane, and nucleocapsid). The root-to-tip distances, the estimated evolutionary rates, and the time-scaled phylogenies of virus strains were determined using the TreeTime (Sagulenko et al. 2018). In addition, we also used BEAST software v2.7.3 (Bouckaert et al. 2014) to estimate the evolutionary rate with a strict clock model and Bayesian skyline coalescent. Sequences of vaccine strains were not included in the evolutionary dynamic analysis. Besides, a coalescent-based nonparametric skygrid prior was used to estimate the effective population size over time (Gill et al. 2013) also in BEAST software. Chains length was set to 800 million and convergence was examined using the Tracer software v1.7 (Rambaut et al. 2018) with a burn-in period of 10% of the total chain length. All parameters estimated using BEAST software yielded an effective sampling size (ESS) >200.

### Recombination Analysis

In addition to PEDV strains, five other coronaviruses sequence were also included in the recombination analysis (supplementary table S4, Supplementary Material online). The strains of four geographical regions (China, South Korea, the US, and Europe) with their corresponding vaccine strains were included in the recombination analysis. Phylogenetic networks (NeighborNet) were constructed by SplitsTree (Huson and Bryant 2006). The recombination events in viral genomes were detected using the recombination detection program 5 (RDP5) as described previously (Martin, Varsani, et al. 2021). Only the events that passed three out of seven implemented methods in RDP5 with a *P*-value cutoff of 0.05 were considered true recombination events. The recombinant sequences were then removed, and the program was repeated until no more recombination events were detected. Potential recombination events were further verified and visualized by using the SimPlot Program v3.5.1 (http://sray.med.som.jhmi.edu/RayAoft/Simplot/).

### Haplotype Dynamic Analysis

All 672 nucleic acid sequences of PEDV non-structural protein 5, 8, and 12 (NSP5, NSP8, and NSP12) were extracted from the aligned PEDV complete genomes, and the haplotypes were obtained using the DnaSP6 software (Rozas et al. 2017). The Templeton, Crandall, and Sing network of all NSP8 haplotypes was constructed using the POPART software (Templeton et al. 1992; Leigh and Bryant 2015) followed by manual adjustment using the Cytoscape v3.8.2 (Smoot et al. 2011).

### Time-Calibrated Phylogeny Reconstruction and Phylogeographic Analysis of PEDV

IQ-Tree was employed to find the best nucleotide substitution model based on Bayesian information criterion values (Nguyen et al. 2015) and the best fitting substitution model was GTR + G5 + I. Bayesian evolutionary and phylogeographic analyses were performed using the BEAST software v2.7.3 and BEAGLE library v2.167 (Ayres et al. 2012) was employed to increase computational performance. The site model used for all BEAST analyses was GTR + G5 + I for two different codon partitions (1 + 2, 3). According to the path and stepping-stone sampling, the skygrid coalescent model with an uncorrelated relaxed clock was chosen. Three different runs (random seeds) of 800 million generations converged to similar values. Outputs were analyzed using the Tracer v1.7 (Rambaut et al. 2018) to ensure all parameters had an ESS superior to 200. In the phylogeographic analyses, the sampling country was used as a discrete trait with a total of 21 different discrete locations. The discrete phylogeographic analysis was performed using Symmetric substitution model and infer social network with Bayesian stochastic search variable selection (Lemey et al. 2009). The SpreaD3 v0.9.7 (Bielejec et al. 2016) software was used to visualize the discrete transmission routes and to calculate the Bayes factor (BF). The lognormal RRW model (Lemey et al. 2010) was chosen to perform a continuous phylogeographic analysis and the results were visualized using R package seraphim v 1.0 (Dellicour et al. 2016).

### Protein Structure Analysis

The sequences of the S gene were in silico translated and aligned using the MUSCLE v3.8.31. The structures of S proteins of G1 genotype (CV777, CH/S, GDS09, SD-M, and LZC) and G2 genotype (JSS04, GDS29, PEDV-8C, GDS46, and JSHA2013) strains were homology-modeled using the SWISS-MODEL (https://swissmodel.expasy.org/) and were visualized using the PyMOL (Molecular GraphicsSystem, Version 2.0 Schrödinger, LLC, https://pymol.org/2/).

### Expression and Purification of PEDV S1 Peptide

Using the amino acid sequences of S1 protein of PEDV G1 and G2 strains, 14 peptide segments were generated (supplementary table S8, Supplementary Material online). The sequences of these peptides were codon-optimized for the prokaryotic expression system and were then cloned into the PEX4T-1 vector separately. Fusion peptides were expressed in *Escherichia coli* BL21 through isopropyl-beta-D-thiogalactopyranoside induction for 4 h, and the bacterial solution was centrifuged at 12,000 rpm for 5 min. Precipitation was collected and lysed with an ultrasonic cell disruptor. The fusion peptides were purified by affinity chromatography columns filled with GSTrap FF. Samples obtained in each step were taken and assessed by SDS-PAGE and Western blot.

## Supplementary Material

Supplementary data are available at *Molecular Biology and Evolution* online.

## Supplementary Material

msad052_Supplementary_Data

## Data Availability

All sequenced strains in the study are available in the National Center for Biotechnology Information (NCBI) with accession number listed in supplementary table S3, Supplementary material online.
