# Supplementary material for: Global Dynamics of Porcine Enteric Coronavirus PEDV Epidemiology, Evolution, and Transmission
Source: Mol Biol Evol. 2023 Mar 3;40(3):msad052. doi: 10.1093/molbev/msad052 (PMC10027654; doi:10.1093/molbev/msad052)
Supplement: msad052_Supplementary_Data [file msad052_supplementary_data.zip › Supplementary infomation for all supplementary figures.pdf]

Supplementary Figures and Supplementary Figures legend

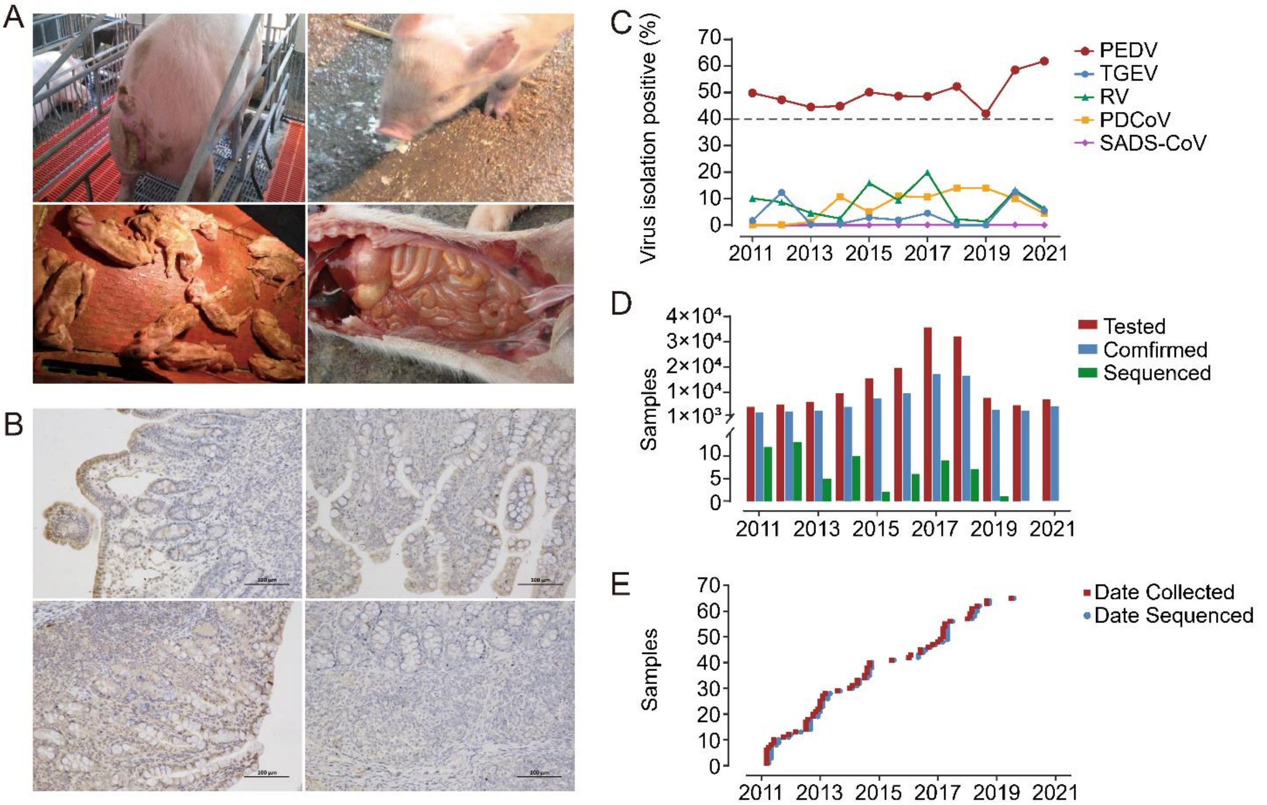

Supplementary Figure 1. Identification, isolation, and sequencing of PEDV. (A) Pigs showing clinical symptoms of PED: feces-stained perineum, vomiting, dead piglets with weight loss, dehydration, and acute watery diarrhea. (B) Intestinal epithelial villi damage of pigs with diarrhea symptoms. (C) The dynamics of a positive rate of five viruses that caused diarrhea symptoms in pigs from 2011 to 2021. (D) The number of samples that were tested, confirmed as PEDV positive, and sequenced in the study. (E) The collection dates and sequenced dates of 65 complete genomes of PEDV.

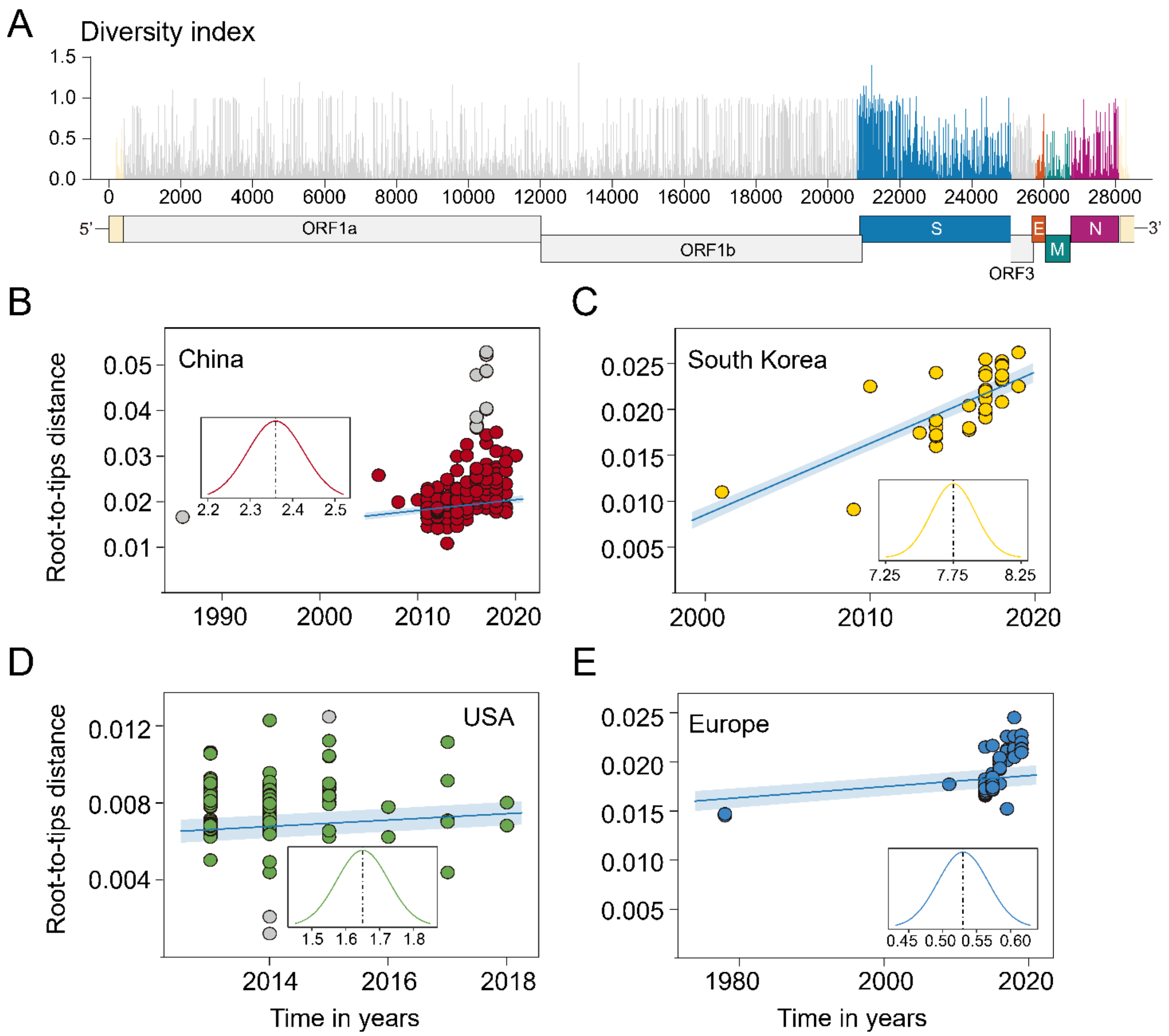

Supplementary Figure 2. (A) Entropy across the entire 672 PEDV genome sequences and (B-E) root-to-tip distance plots on sampling date and the estimated evolutionary rates of PEDV strains of four geographical regions. The strains depicted in grey in the outer plots were excluded in the linear fitting and evolutionary rate estimation according to TreeTime. The inner plots show the posterior probability densities of the mean estimated evolutionary rate and the x-axis represents the evolutionary rate ($\times 10^{-4}$ substitutions per site per year).

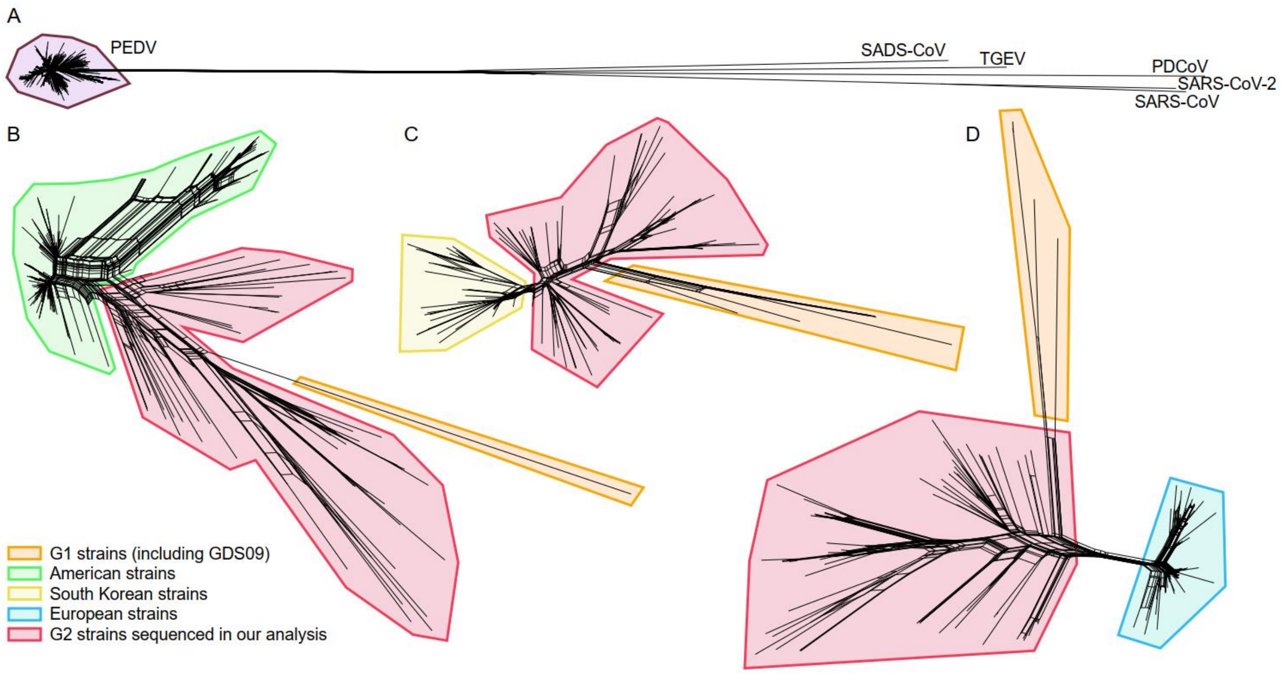

Supplementary Figure 3. Phylogenetic networks (NeighborNet) constructed by SplitsTree based on the complete genome sequences. (A) Network constructed using 672 PEDV and five other coronaviruses' complete genome sequences. PEDV strains are clustered in the purple polygon . SADS-CoV, swine acute diarrhea syndrome coronavirus (Genbank accession No., MF167434); TEGV, transmissible gastroenteritis virus (Genbank accession No., DQ811789); PDCoV, porcine deltacoronavirus (Genbank accession No., KJ481931); SARS-CoV, severe acute respiratory syndrome coronavirus (Genbank accession No., NC_004718); SARS-CoV-2, severe acute respiratory syndrome coronavirus 2 (Genbank accession No., MT121215). (B) Network constructed using 236 PEDV strains from USA and 65 PEDV strains sequenced in our study. G1 strain (GDS09 only) was highlighted in the orange polygon and USA strains were clustered in the green polygon. (C) Network constructed using 42 PEDV strains from Korea and 65 PEDV strains sequenced in our study. G1 strain from Korea and GDS09 was highlighted in the orange polygon and Korean strains were clustered in the yellow polygon. (D) Network constructed using 90 PEDV strains from Europe and 65 PEDV strains sequenced in our study. G1 strain from Europe and GDS09 was highlighted in the orange polygon and European strains were clustered in the blue polygon. The 65 PEDV strains sequenced in our study were clustered in red polygons.

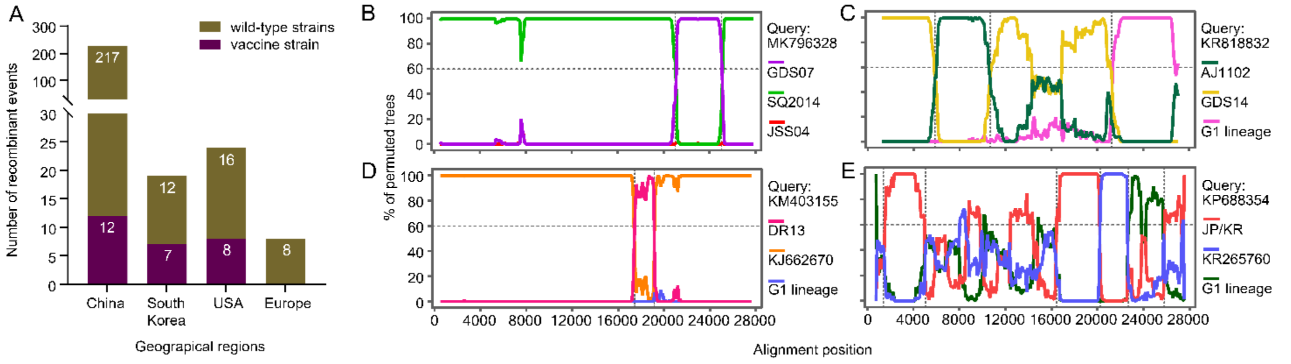

Supplementary Figure 4. Recombination features of wild-type and vaccine PEDV strains. (A) Numbers of recombination events detected in wild-type and vaccine PEDV strains isolated from indicated geographical regions. (B-E) A rescaled structure of four PEDV genomes and bootscanning recombination analysis based on variable genomic sites. The y-axis shows the percentage of permutated trees using a sliding window of 200 bp width centered in the plotted position, with a step size of 20 bp. The dashed line indicates 60% bootstrap support. Different colored lines represent different strains. (B) Strain CN/Liaoning25/2018 (Genbank accession No., MK796238) from China was used as query strain and strains GDS07 (Genbank accession No., MH726370; dark purple), vaccine strain SQ2014 (Genbank accession No., KP728470; green), and JSS04 (Genbank accession No., MW143083; red) were used as putative parental strains. (C) Strain XY2013 (Genbank accession No., KR818832) from China was used as query strain and strains vaccine strain AJ1102 (Genbank accession No., JX188454; green), GDS14 (Genbank accession No., MH726393; yellow), and G1 lineage strains (light pink) were used as putative parental strains. (D) Strain KNU-1406-1 (Genbank accession No., KM403155) from Korea was used as query strain and strains vaccine strain DR13 (Genbank accession No., JQ023161; dark pink), KNU-1305 (Genbank accession No., KJ662670; light orange), and G1 lineage strains (light purple) were used as putative parental strains. (E) Strain Hawaii/39249/2014 (Genbank accession No., KP688354) from the USA was used as query strain and strains vaccine strain JP/KR (Genbank accession No., MC425617/LY411674; dark orange), USA/Minnesota211/2014 (Genbank accession No., KR265760; light purple), and G1 lineage strains (green) were used as putative parental strains.

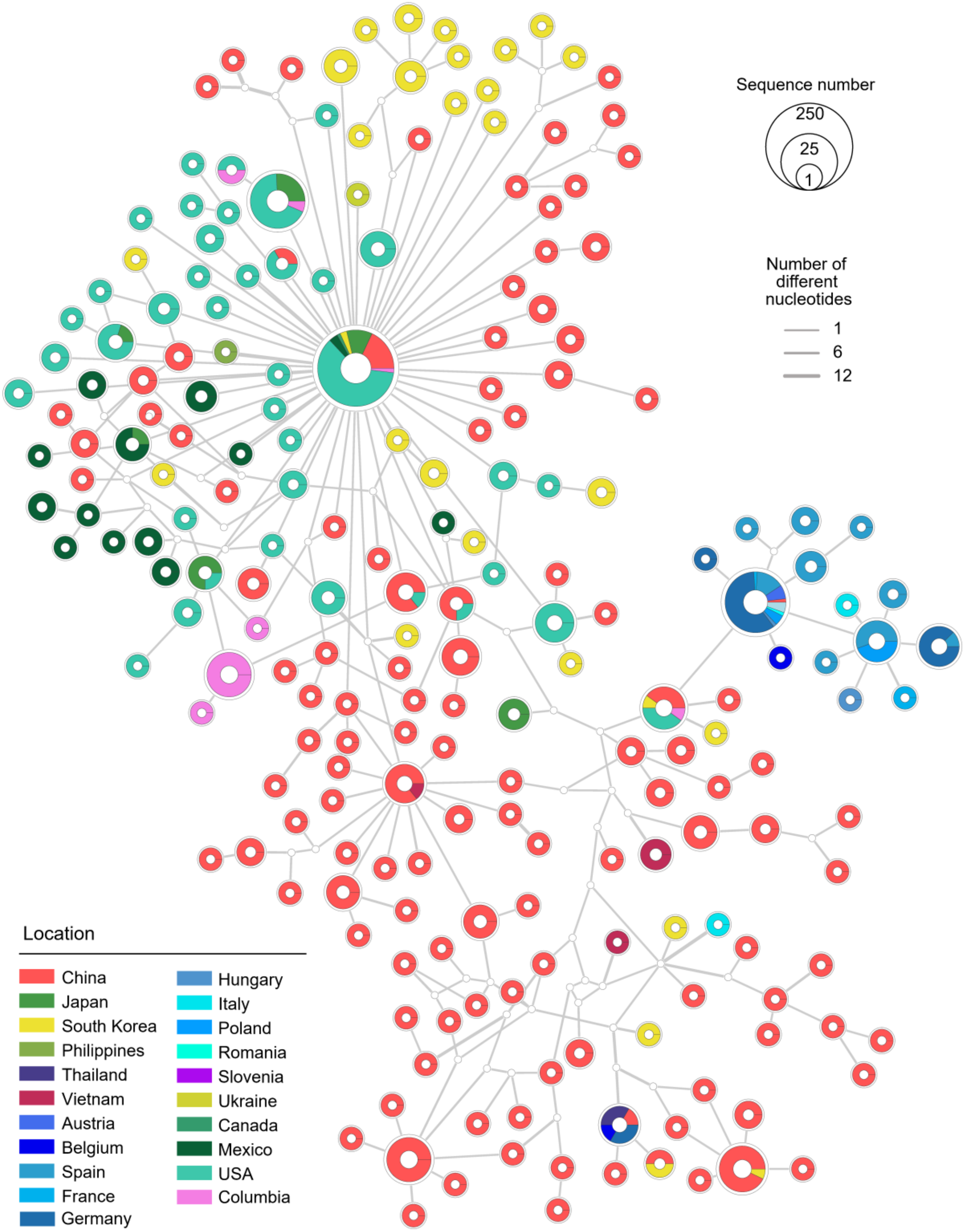

Supplementary Figure 5. A median-joining haplotype network constructed from an alignment of 672 sequences of NSP5 of PEDV genome. The median-joining haplotype network covered the 212 multiple viral haplotypes. Each circle represents a unique haplotype, and its size represents the number of haplotypes. The different colors code the respective geographical distribution. The size of edges represents the number of different nucleotides between two haplotypes.

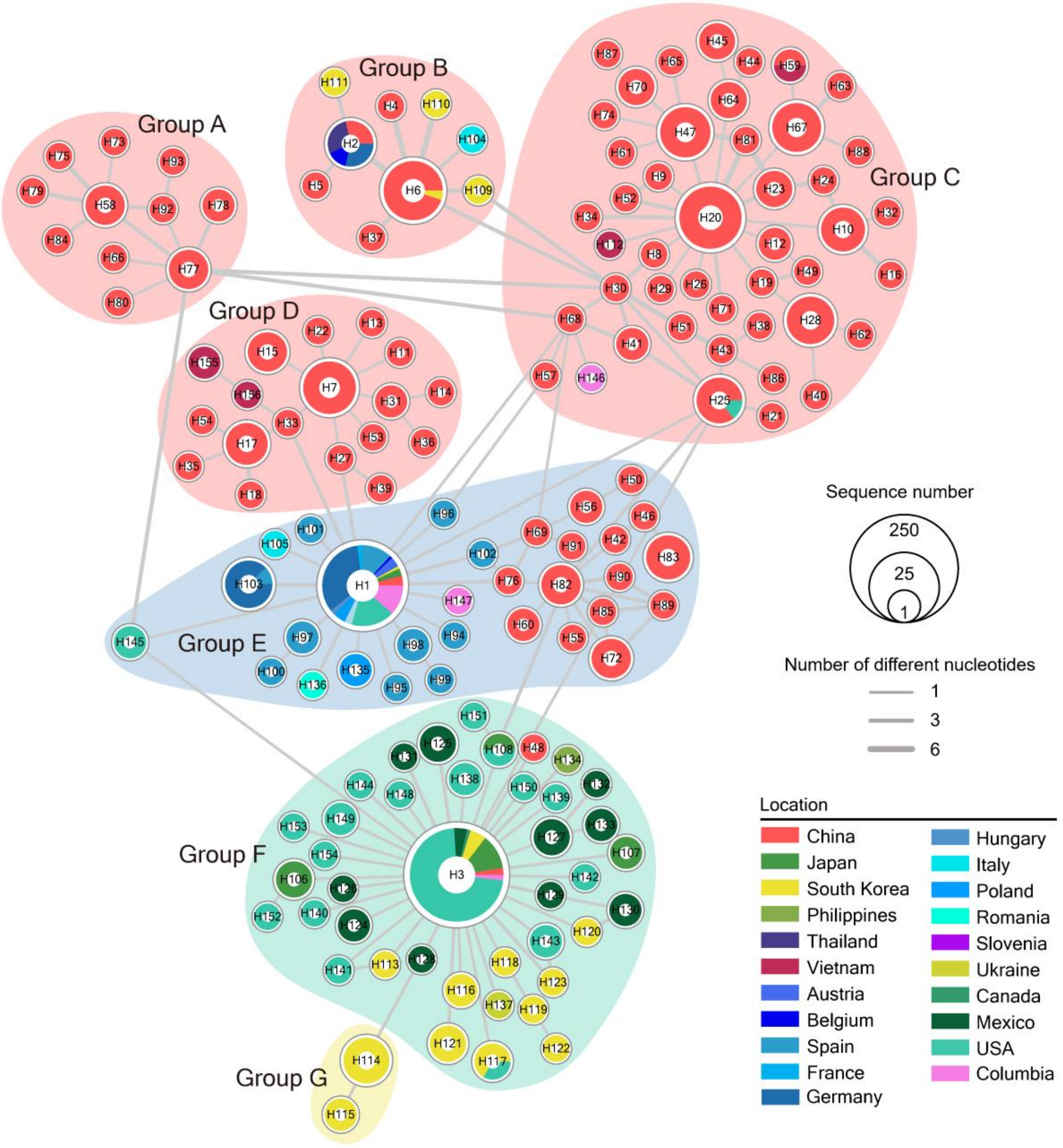

Supplementary Figure 6. A median-joining haplotype network constructed from an alignment of 672 sequences of NSP8 of PEDV genome. The median-joining haplotype network covered the 156 multiple viral haplotypes. Each circle represents a unique haplotype, and its size represents the number of haplotypes. The different colors code the respective geographical distribution. The size of edges represents the number of different nucleotides between two haplotypes. Seven groups of haplotypes (A~G) were identified in our study.

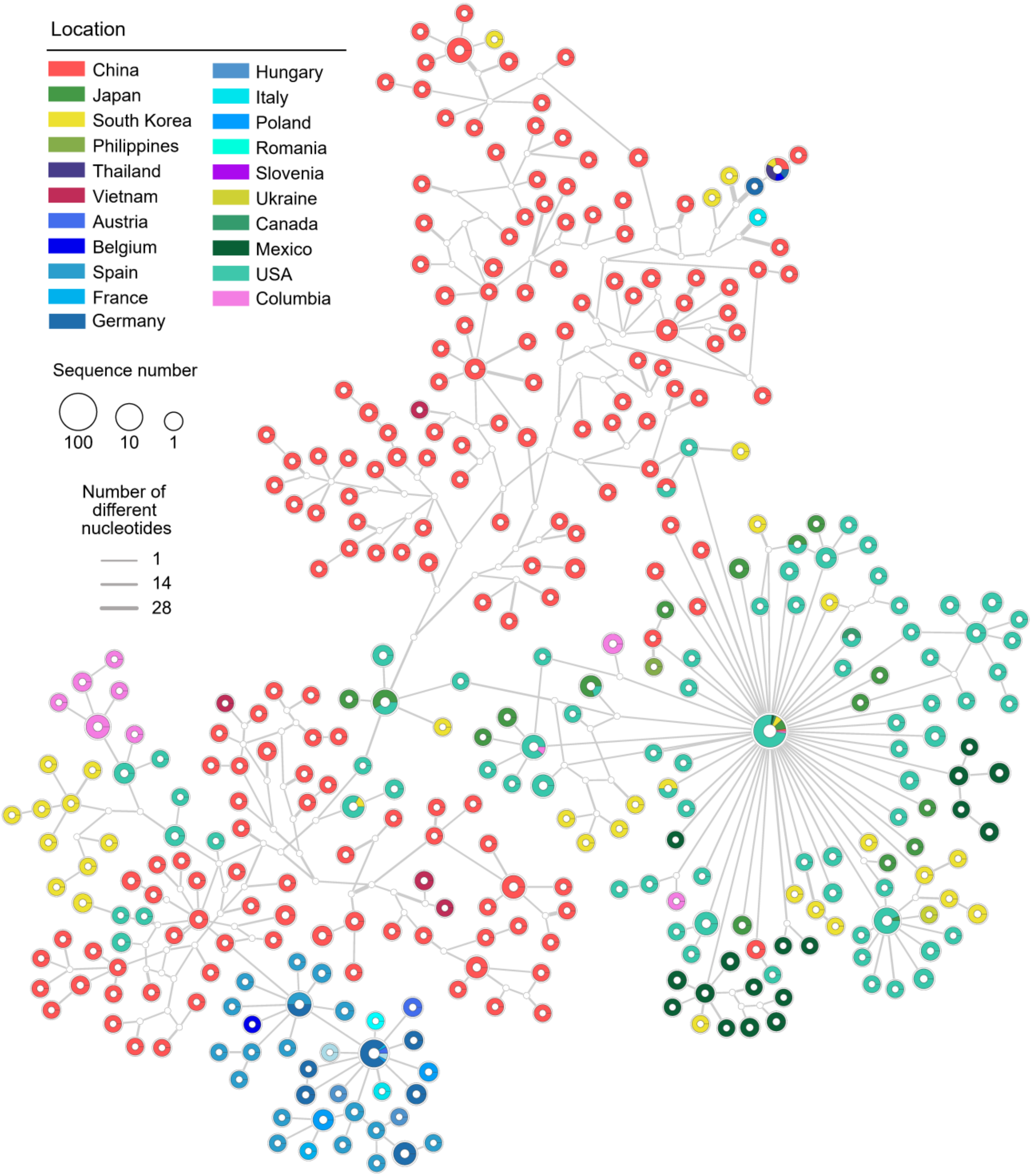

Supplementary Figure 7. A median-joining haplotype network constructed from an alignment of 672 sequences of NSP12 of PEDV genome. The median-joining haplotype network covered the 356 multiple viral haplotypes. Each circle represents a unique haplotype, and its size represents the number of haplotypes. The different colors code the respective geographical distribution. The size of edges represents the number of different nucleotides between two haplotypes.

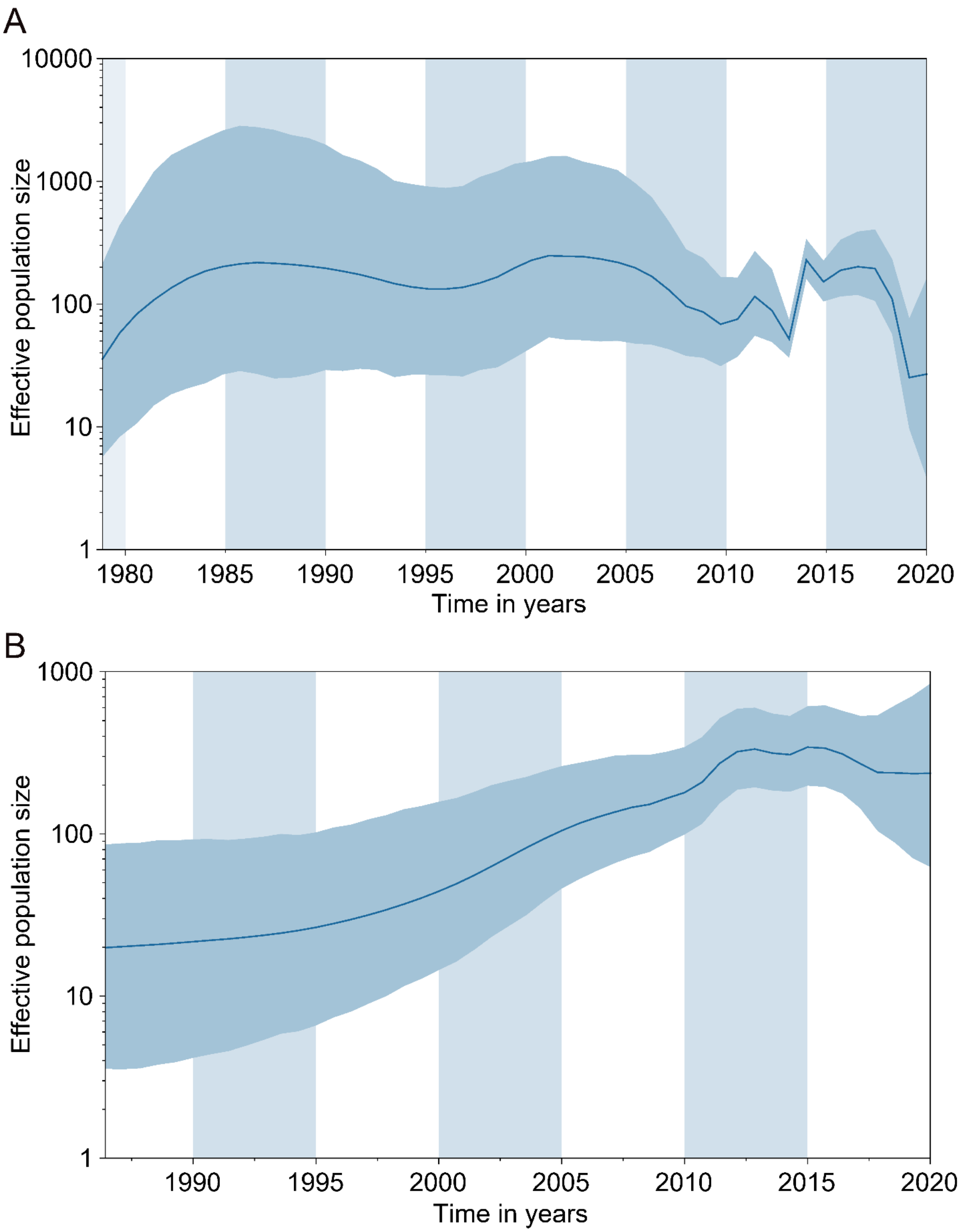

Supplementary Figure 8. The effective population size (N_e) of PEDV (A) global (n = 672) and (B) Chinese dataset (n = 237). The blue solid line indicates the median estimate and the blue area indicates the 95% confidence interval (CI).

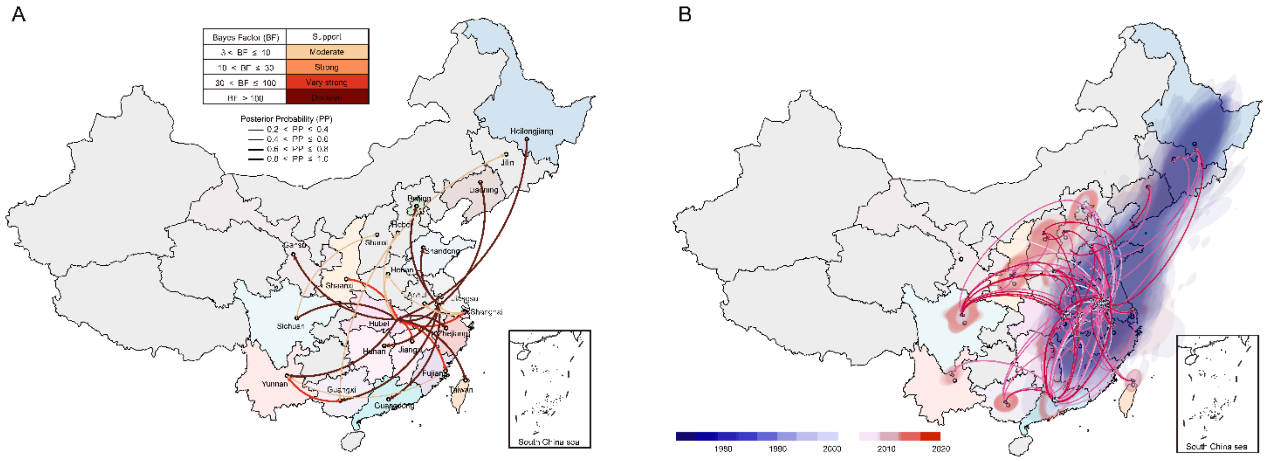

Supplementary Figure 9. Spatiotemporal diffusion of Chinese PEDV strains. (A) The discrete phylogeographic analysis was performed with the Bayesian stochastic search variable selection (BSSVS) approach. We displayed the intensity of the estimated transition events associated with a bayes factor (BF) support higher than 3. The thickness of the dissemination links represented the posterior probability (PP). (B) Continuous phylogeographic analyses were performed with the lognormal RRW diffusion model. For the continuous reconstruction, we mapped the MCC tree and 95% highest posterior density regions based on trees subsampled from the post-burn-in posterior distribution of trees. Nodes of the tree are colored according to a color scale ranging from blue (tMRCA) to red (most recent sampling time). A 95% highest posterior density regions were computed for successive time layers, superimposed using the same color scale reflecting time cropped using Chinese international borders. On both maps, subnational Chinese province borders are represented by grey lines.

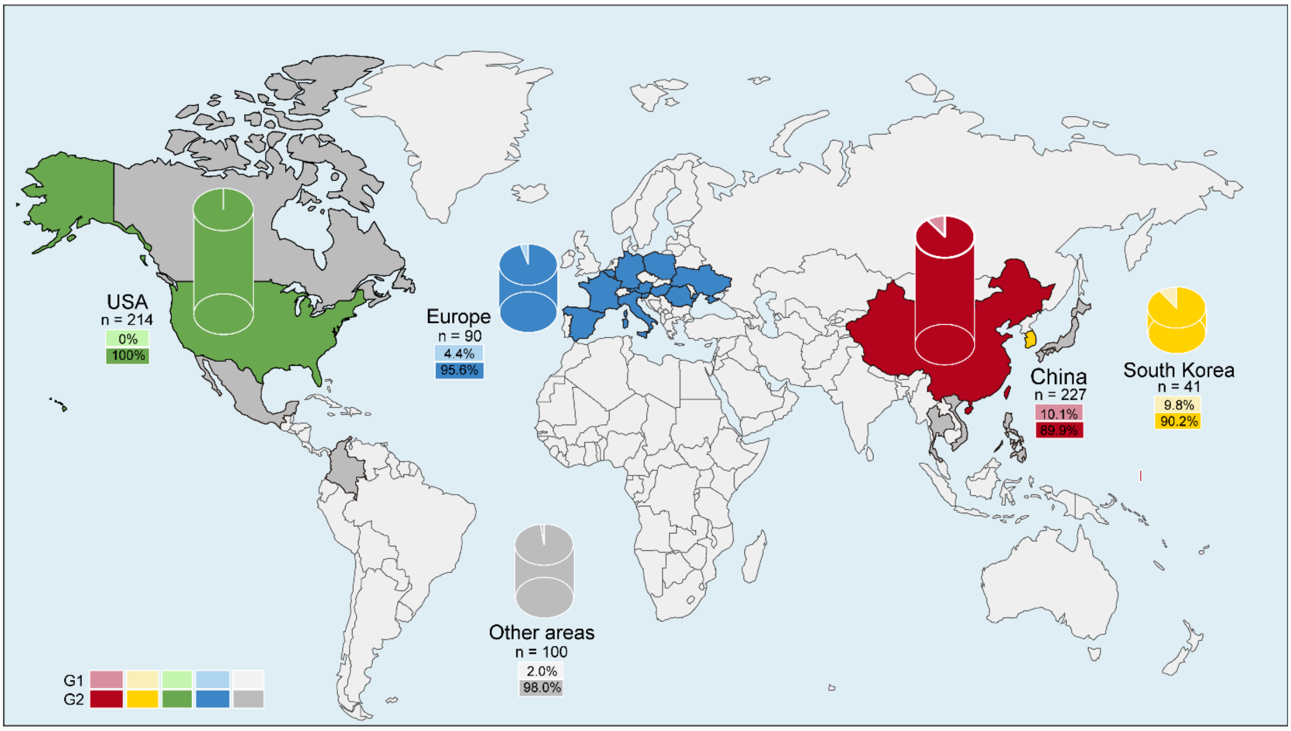

Supplementary Figure 10. The genotypes distribution of 672 PEDV strains. The proportion of G1 strains is shown in lighter shades of the given colors. The proportion of G2 strains is shown in darker shades of the given colors. Green indicates the USA, blue indicates Europe, red indicates China, Yellow indicates South Korea, and grey indicates other geographical regions.

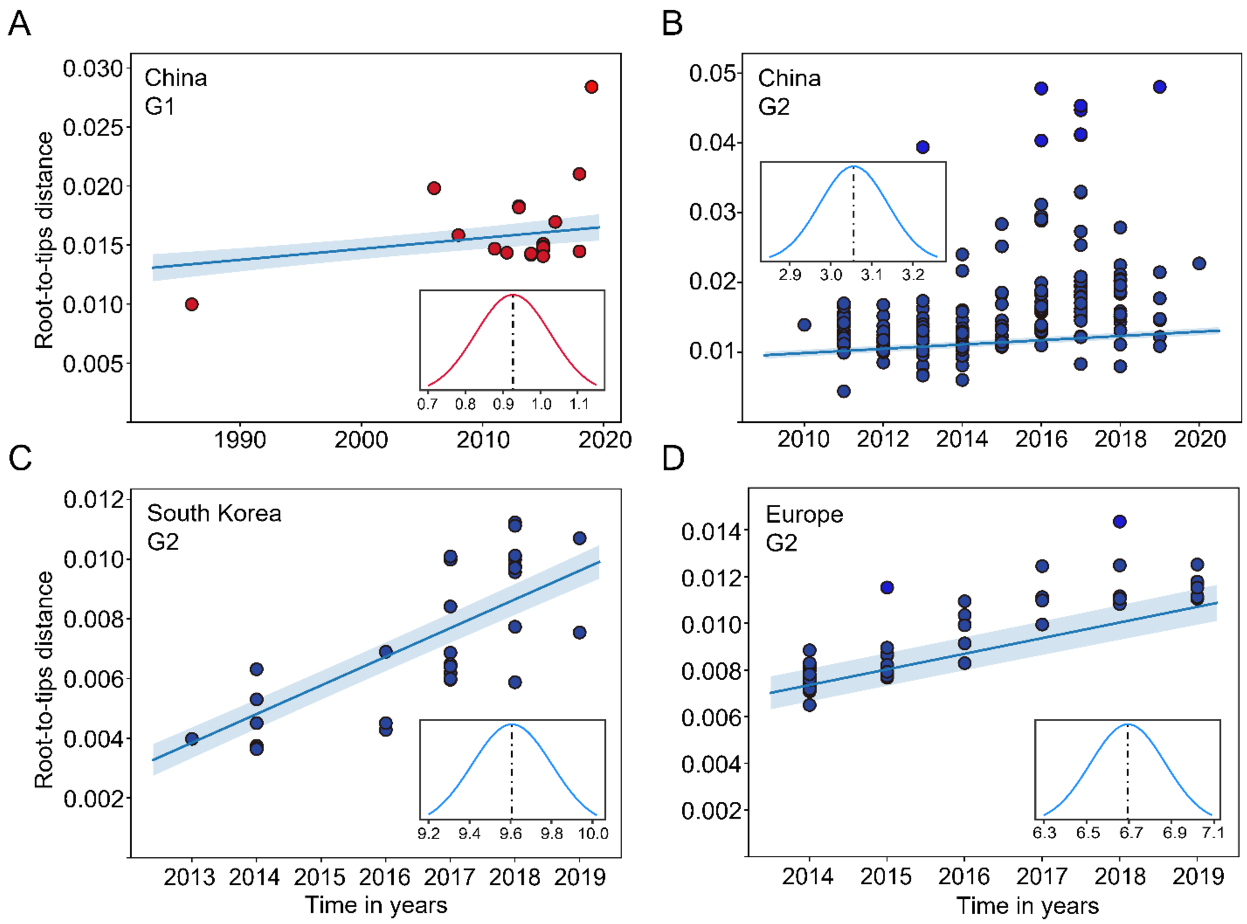

Supplementary Figure 11. Root-to-tip distance plots on sampling date and the estimated evolutionary rates of PEDV strains. (A) Chinese strains (*n* = 20) of G1 genotype. (B) Chinese strains (*n* = 207) of G2 genotype. (C) Korean strains (*n* = 37) of G2 genotype. (D) European strains (*n* = 86) of G2 genotype. The strains depicted in grey in the outer plots were excluded in the linear fitting and evolutionary rate estimation according to TreeTime. The inner plots show the posterior probability densities of the mean estimated evolutionary rate and the x-axis represents the evolutionary rate ($\times 10^{-4}$ substitutions per site per year).

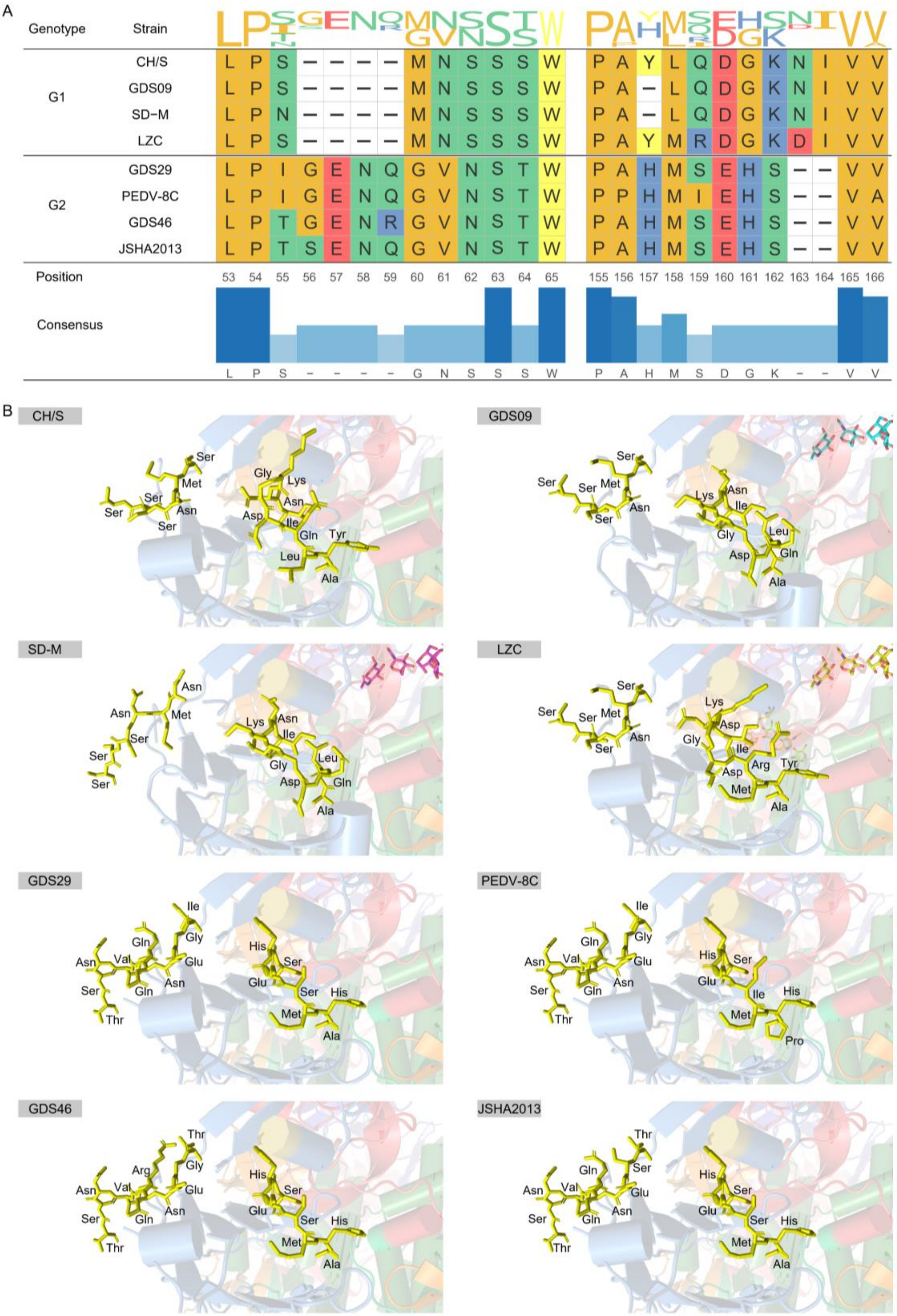

Supplementary Figure 12. Identification of amino acid mutation sites in the two regions (at alignment position 53~65 and 155~166) of S protein from G1 and G2 strains. (A) The alignment. (B) Cartoon representation of amino acid sequences of the spike protein of strains. G1 strains included CH/S (Genbank accession No., JN547228), GDS09 (Genbank accession No., MH726408), SD-M

(Genbank accession No., JX560761) and LZC (Genbank accession No., EF185992). G2 strains included GDS29 (Genbank accession No., MH726377), PEDV-8C (Genbank accession No., KM609205), GDS46 (Genbank accession No., MH726402) and JSHA2013 (Genbank accession No., KR818833).
